# Pregnancy Outcomes in Chinese Patients with Systemic Lupus Erythematosus (SLE): A Retrospective Study of 109 Pregnancies

**DOI:** 10.1371/journal.pone.0159364

**Published:** 2016-07-21

**Authors:** Ming Ku, Shuiming Guo, Weifeng Shang, Qing Li, Rui Zeng, Min Han, Shuwang Ge, Gang Xu

**Affiliations:** Division of Nephrology, Department of Internal Medicine, Tongji Hospital, Tongji Medical College, Huazhong University of Science and Technology, Wuhan, Hubei, China; Oklahoma Medical Research Foundation, UNITED STATES

## Abstract

Systemic lupus erythematosus (SLE) is a multisystem autoimmune disease that primarily affects women during their reproductive years. The interaction between SLE and pregnancy remains debated. The objective of this study was to analyze the fetal and maternal outcomes of Chinese women with SLE. A total of 109 pregnancies in 83 SLE patients from June 2004 to June 2014 at a tertiary university hospital were reviewed retrospectively. Patients’ characteristics, clinical and laboratory data during pregnancy were obtained from electronic medical records. After exclusion of elective abortions, the live birth rate was 61.5%. Significantly, APS (antiphospholipid syndrome), disease activity, hypertension, hypocomplementemia, thrombocytopenia, and anemia during pregnancy were more commonly observed in fetal loss pregnancies than in live birth pregnancies. Compared to the 64 women with a history of SLE, 19 women with new-onset lupus during pregnancy had worse pregnancy outcome. Furthermore, the 64 patients with a history of SLE were divided into lupus nephritis group and SLE group (non-renal involvement). We found that the lupus nephritis group had worse maternal outcome than the SLE group. We conclude that new-onset lupus during pregnancy predicts both adverse maternal and fetal outcomes, while a history of lupus nephritis predicts adverse maternal outcomes. It is essential to provide SLE women with progestational counseling and regular multispecialty care during pregnancy.

## Introduction

Systemic lupus erythematosus (SLE) is a complex, multisystem disease that mainly attacks fertile women. Women with lupus disease are not less fertile than unaffected women, but SLE may increase pregnancy complications, including spontaneous abortion, premature delivery, intrauterine growth restriction (IUGR), and preeclampsia[1]. Although recent studies have shown favorable outcomes[2–6] such as the improved survival rate and the timing of conception after SLE remissive from longer than 6 months to 4 months[7], SLE remains a disease associated with significant fetal and maternal complications[8–10]. The interaction of SLE and pregnancy has been studied but still under debate.

Several studies suggested that renal function and the presence of proteinuria at the time of conception may contribute to adverse maternal and fetal outcomes[7,11–13]. However, some reports showed that the women with a history of lupus nephritis can also have successful pregnancy outcomes[14–17]. Furthermore, new-onset of SLE can even occur during pregnancy[13,18,19]. A study suggested that new-onset SLE during pregnancy generally occurred during the second trimester, with a higher rate of renal and blood involvement[20]. However, there are limited researches associated with the new-onset of SLE during pregnancy and its impacts on the pregnancy outcomes.

In this study, we aimed to study the maternal and fetal outcomes of new-onset SLE and lupus nephritis during pregnancy in Chinese SLE women.

## Patients and Methods

We retrospectively reviewed the medical records of 109 consecutive pregnancies in 83 SLE patients from a tertiary hospital between 2004 and 2014. All patients met at least four of the 1997 revised American College of Rheumatology criteria for the classification of SLE[21]. Those terminated electively were not included in this analysis. Patients with renal insufficiency during pregnancy were also excluded from our study. This study complied with the Declaration of Helsinki Principles and was approved by the Ethics Committee of Tongji Hospital, Huazhong University of Science and Technology. Written informed consent was obtained from each patient.

Medical records were reviewed retrospectively; demographic data, clinical and laboratory information were collected. Laboratory data, including hemoglobin, red and white blood cell and platelet counts, urinalysis, 24-h urine protein excretion, serum creatinine, blood urea nitrogen, albumin, glucose, and uric acid as well as immunologic parameters including complement 3 (C3), complement 4 (C4), immune globulin, Coomb’s test, antinuclear antibodies (ANA), anti-ds DNA antibodies (anti-dsDNA), anti-Smith antibldy, anti-Ro/SSA antibodies, anti-La/SSB antibodies, anticardiolipin antibody (aCL), and anti-β2 glycoprotein 1 antibody.

Patients were divided into new-onset SLE group who were firstly diagnosed with SLE during pregnancy or puerperium and pre-existing SLE group who had a history of SLE. The pre-existing SLE group was further divided into lupus nephritis groups and non-renal involvement SLE group. Lupus nephritis was define as the presence of active urinary sediment and/or proteinuria >0.5 g/day with or without elevation in serum creatinine before 20 weeks gestation. Before ascribing proteinuria, preeclampsia and HELLP (hemolysis, elevated liver enzymes, and low platelets) syndrome had been excluded. SLE disease activity was evaluated according to the SLE Disease Activity Index 2000 (SLEDAI-2K)[22].

Maternal outcomes included SLE flares, disease activity, hypertension, preeclampsia, thrombocytopenia and anemia. Anemia was defined as hemoglobin<100 g/L during pregnancy.

Fetal outcomes for live birth included fetal loss, prematurity, 1-min Apgar scores and intrauterine growth retardation. Fetal loss was the loss of pregnancy due to spontaneous abortion or stillbirth. Gestational age of fetal loss or delivery was also recorded. Spontaneous abortion was defined as the spontaneous loss of a fetus before 20 weeks of gestation, stillbirth as the death of the fetus in utero after 20 weeks of gestation, and elective abortion as the voluntary termination of pregnancy. Preterm birth was a live birth between 23 and 37 weeks and intrauterine growth retardation (IUGR) was defined as birth weight less than 2500g.

### Statistical analysis

Statistical analysis was performed with SPSS version 20. The categorical variables were analyzed by using chi-square test or Fisher’s exact probability test as appropriate. The continuous variables were analyzed by using Student’s t test as appropriate. A value of p<0.05 was regarded as statistically significant.

## Results

### Patient characteristics

A total of 83 patients had 109 pregnancies during our study. As shown in Table 1, the mean age of the patients when diagnosed with SLE was 21.38 ± 5.90 years (range, 9 to 40 years), and the mean age at the time of conception was 28.16 ± 4.96 years (range, 19 to 41 years). The mean SLE duration was 4.26±3.89 years (range, 0 to 21 years). The mean SLEDAI-2K scores at conception was 10.05±6.96. Among these patients, 63 patients were nulliparous and 20 patients were multiparous. Fifteen patients had two pregnancies, four patients had three pregnancies, and one patient had four pregnancies.

**Table 1:** Patients’ characteristics during pregnancy.

Characteristic	Value or no. (%) of patients (n = 83)
Age at disease onset (yr, mean±SD, range)	*21*.*38± 5*.*90 (9–40)*
Age at conception (yr, mean±SD, range)	28.16± 4.96 (19–41)
Disease duration (yr, mean±SD, range)	4.26± 3.89 (0–21)
First pregnancy	63 (75.9%)
Second pregnancy	15 (18.1%)
Third or more pregnancy	5 (6.0%)
Cesarean section	*50 (60*.*24%)*
SLEDAI-2K at conception (score, mean±SD, range)	10.05±6.96 (0–33)
New onset SLE	19 (22.9%)
Neonatal lupus	2 (3%)
Neonatal heart disease	2 (3%)
APS	4 (4.8%)
**Laboratory features at onset of pregnancy**	
ANA	83 (100%)
Anti-dsDNA	72 (86.7%)
Anti-Ro/SSA	55 (66.2%)
Anti-La/SSB	24 (28.9%)
Anti-Sm	27 (32.5%)
aCL	10 (12%)
Hypocomplementania	56 (67.5%)
C3	25 (30.1%)
C4	40 (48.2%)
**Drugs taken at the onset of pregnancy**	
prednisone	57 (68.7%)
Aspirin	10 (12%)
Azathioprine	5 (6%)
Cyclophosphamide	0
Methotrexate	0
chloroquine	30 (36.1%)

SLEDAI, systemic lupus erythematosus disease activity index; Ab, antibodies; aCL, Anticardiolipin antibody.

Autoantibodies during pregnancy were positive as follows: anti-ds DNA in 72 patients (86.7%), anti-Ro/SSA in 55 patients (66.2%), anti-La/SSB in 24 patients (28.9%), and anti-Sm in 27 patients (32.5%). Hypocomplementemia was observed in 56 patients (67.4%). Four patients were diagnosed as antiphospholipid syndrome (APS) during pregnancy and one mother’s baby died in utero during the 26^th^ week.

Of the 83 women during pregnancy, 57 (68.7%) were treated with low-dose glucocorticoid, with an average prednisone dose of 5 mg/d (range 2.5–15 mg). A total of 30 patients (36.1%) were taking hydroxychloroquine (HCQ) (200 mg/d). Flares were treated with prednisone (0.5-1mg/kg/d). Aspirin (100mg/d) was prescribed for patients positive for aCL antibodies.

### Pregnancy outcomes and factors associated with adverse pregnancy outcome

Fetal loss (including spontaneous abortion and stillbirth) occurred in 42 pregnancies (38.5%), and the live birth pregnancies (including premature births, live birth at full term and postmature births) occurred in 67 pregnancies (61.5%) (Table 2). There were two neonatal lupus and two fetal heart malformations (one tetralogy of Fallot, and one atrial septal defect). The neonatal with tetralogy of Fallot died at the second day after birth. APS, disease activity at conception, thrombocytopenia, hypocomplementemia, anemia, and hypertension were significantly associated with fetal loss at univariate analysis, while binary logistic regression analysis showed APS, disease activity at conception, thrombocytopenia, anemia, and hypertension were significant predictors of fetal loss.

**Table 2:** Comparison of clinical features between fetal loss pregnancies and live birth pregnancies.

Clinical features	Fetal loss pregnancies (n = 42 cases)	Live birth pregnancies (n = 67 cases)	Univariate analysis p	Logistic regression analysis p
APS	4 (9.5%)	0	<0.001	0.012
Hypocomplementemia	35 (83.3%)	22 (32.8%)	0.001	-
SLEDAI-2K at conception (mean±SD, range)	14.9±7.8	8.0±5.5	<0.001	0.002
Anti-dsDNA	37 (88.1%)	60 (89.6%)	1.0	-
Proteinuria	34 (80.1%)	41 (61.2)	0.13	-
Thrombocytopenia	21 (50%)	12 (17.9%)	0.01	0.02
Hypertension	21 (50%)	16 (23.9%)	0.04	0.001
Anemia	34 (80.1%)	23 (34.3%)	0.04	0.007
Lupus Nephritis	16 (38.1%)	21 (31.3%)	0.59	-

Pregnancies loss include spontaneous abortion (<12 weeks), interuterine fetal death (≥12 weeks) and perinatal deaths. Live birth pregnancies include premature births(≥22 weeks, <37 weeks) and full-term births (≥37 weeks, <42 weeks). SLEDAI, systemic lupus erythematosus disease activity index.

### Adverse pregnancy outcomes between new-onset SLE and pre-existing SLE patients

Of the 83 patients, 19 (22.9%) women were firstly diagnosed with SLE during pregnancy or puerperium, and 64 (77.1%) women were pre-existing SLE. Among the 19 new-onset SLE patients, 12 patients had SLE that first occurred during the second trimester (63.1%), mainly during the 18^th^ week. Compared to pre-existing SLE pregnancies, patients with new-onset SLE during pregnancy had a significantly higher fetal loss (73.7% vs 15.6%, p<0.01) (Table 3). Disease activity, such as high SLEDAI scores and hypocomplementemia were commonly founded among new-onset SLE patients during pregnancy. The levels of hemoglobin and neonate Apgar score (1 min after birth) were significantly lower in the new-onset SLE pregnancies and the rate of lupus nephritis was significant higher in new-onset SLE group. The remaining manifestations were not significant differences between the two groups. All the patients in pre-existing SLE group were still alive after delivery. Unfortunately, one patient of the new-onset SLE group died during pregnancy.

**Table 3:** Comparison of pregnancy outcomes between new onset SLE and pre-existing SLE patients.

	New-onset SLE (n = 19 cases)	Pre-existing SLE (n = 64 cases)	p
**Maternal outcome**			
SLEDAI-2K at conception	15.4±7.4	8.4±5.9	<0.001
Hypocomplementemia	12 (63.2%)	18 (28%)	0.01
Thrombocytopenia	7 (36.9%)	14 (22.6%)	0.21
Anemia	15 (78.9%)	24 (37.5%)	0.01
Hypertension	8 (42.1%)	16 (18.4%)	0.16
Proteinuria	16 (88.9%)	35 (59.3%)	0.04
Lupus nephrities	12 (66.7%)	21 (40.4%)	0.05
Maternal death	1 (5.3%)	0	0.07
**Fetal outcome**			
Fetal loss	14 (73.7%)	10 (15.6%)	<0.01
Premature (22–37 W)	3 (20%)	18 (28.6%)	0.5
Apgar score 1-min	3.6±4.17	7.4±2.8	0.01
IUGR	11 (57.9%)	25 (39.1%)	0.15
Neonatal lupus	0	2 (3.1%)	0.44
Neonatal heart disease	0	2 (3.1%)	0.44

IUGR, intrauterine growth retardation.

### Pregnancy outcomes in non-renal involvement SLE group and lupus nephritis group

The 64 pre-existing SLE patients were divided into two groups by kidney impairment. We found that lupus nephritis pregnancies have more maternal adverse outcomes, including proteinuria, hypertension, disease activity and SLE flares, compared to SLE group. However, the fetal outcome between the two groups was not significantly different (Table 4).

**Table 4:** Comparison of pregnancy outcomes between SLE group and lupus nephritis group.

Pregnancy outcome	Non-renal involvement SLE group (n = 35 cases)	Lupus nephritis (n = 29 cases)	p
**Maternal outcome**			
SLEDAI-2K	6.5±5.4	10.5±5.9	0.009
SLE flares	12 (34.3%)	18 (62.1%)	0.001
UA	316±118	360±114	0.13
Proteinuria	13 (37.1%)	22 (75.9%)	0.005
ALB	33.3±4.3	31.2±6.3	0.14
Anemia	11 (31.4%)	13 (44.8%)	0.27
Thrombocytopenia	4 (11.4%)	10 (34.5%)	0.30
Hypertension	5 (14.3%)	11 (37.9%)	0.025
Hypocomplementania	8 (22.9%)	10 (34.5%)	0.52
Serum creatinine	49.7±20.3	58.6±27.5	0.43
**Fetal outcome**			
Fetal loss	3 (8.6%)	6 (20.7%)	0.17
Premature	9 (25.7%)	9 (31.0%)	0.15
IUGR	10 (28.6%)	9 (31.0%)	0.78
Apgar score (mean±SD)	7.6±2.7	7.4±2.4	0.72
Neonatal lupus	2(5.7%)	0	0.18
Neonatal heart disease	2(5.7%)	0	0.18

IUGR, intrauterine growth retardation; ALB, albumin; UA, uric acid.

## Discussion

Recent studies suggest progress in understanding the association between pregnancy and SLE and better treatment choices. Pregnancy outcomes have become more favorable during recent years. However, poor mother outcomes and fetal loss remains higher than in the general population. In addition, new-onset of SLE during pregnancy is still very common but reported scarcely. Published reports on pregnancy outcomes in Chinese women with SLE and lupus nephritis have some limitations. For example, Tian’s research group demonstrated the related factors of fetal loss but not reported the maternal outcomes in Chinese women with SLE[23]. Liu’s research group demonstrated the adverse outcome between active and inactive SLE pregnancies, however, they didn’t focus on the difference of outcome between pre-existing SLE pregnancies and new-onset SLE during pregnancy, LN pregnancies and non-renal involvement SLE pregnancies[24]. In our study, we retrospectively reviewed 83 SLE patients of 109 pregnancies during the last decade in our university hospital in China. After excluding elective abortions, our live birth rate was 61.5%, which is lower than the 80.5%-91.9% reported in previous studies[4–6], and our fetal loss of 38.5% is high compared to previous retrospective reports of 8%-40%[24–26]. The low live birth rate can be explained by the reasons below: On one hand, our hospital is a major tertiary comprehensive center having a lot of patients with more severe and complicated disease. Our hospital may represent more than 800 tertiary hospitals in China. On the other hand, a high proportion of new-onset SLE patients during pregnancies who have lower birth rate. When the new-onset SLE pregnancies group is excluded, the live birth rate is 84.4%.

Hydroxychloroquine has been used extensively for SLE where it has a good reputation for controlling the dermatological complications in SLE in recent years. Our prevalence of hydroxychloroquine use was 36%, which is lower than 55% in the US[27]. However, it is similar to that hydroxychloroquine prescribed from 18% to 32.2% in Spain[28]. There are several reasons below: Firstly, our patients ranged from January 2004 to January 2014. In the previous few years, hydroxychloroquine was not widely used in our country. Secondly, hydroxychloroquine was prescribed more frequently by rheumatologists than by primary care physicians or nephrologists in China. Thirdly, the long disease course and the tough economic condition of SLE patients may cause poor compliance. Our study suggests the importance for both rheumatologists and nonrheumatologists such as primary care physicians and nephrologists to consider the use of hydroxychloroquine more frequently in their SLE patients.

There was a small proportion of APS patients in our study. Previous studies have demonstrated APS was a major risk for fetal loss and mother health[29,30]. Indeed, in our study one mother with APS lost her baby and then died of bleeding. Studies of the pathogenesis of APS indicate that lupus anticoagulant, anticardiolipin antibody and antiphospholipid antibody play a direct pathogenic role resulting in placental insufficiency[29,31].

In this study, new-onset SLE during pregnancy or puerperium occurred in 19 (22.9%) of our patients, suggesting that first diagnosis of SLE was not a rare event during pregnancy. This group of patients often has poor prognosis. The recognition of new-onset SLE during pregnancy can sometimes be difficult because the symptoms and signs may mimic those of normal pregnancy. Edema, fatigue, hair loss, erythema, mild anemia, decreased albumin concentrations, and headaches frequently accompany normal pregnancy[32,33]. When the condition persists or gets worse, such as severe anemia, thrombocytopenia, and proteinuria, the new-onset SLE pregnancies then referred to a tertiary hospital. In addition, we also found that new-onset lupus primarily occurred during the second trimester. It is reported that estrogens are a predisposing factor for SLE occurrence[34,35]. However, reports about new-onset SLE during pregnancy and its impacts on maternal and fetal health are lacking. Hormonal swings of pregnancy in the second trimester accompanying with the alteration of immune system, may be the risk factor for new onset SLE during pregnancy[36]. Our study demonstrated that new-onset SLE patients have more severe disease with frequent renal and platelet involvement, which is in line with one previous report[20].

The impact of lupus nephritis on maternal and fetal outcomes is controversy. Several previous studies suggested that lupus nephritis may contribute to adverse pregnancy outcomes[13,17,37,38]. In particular, active lupus nephritis leads to severe pregnancy outcomes[12,39–41]. By contrast, some reports showed that the lupus nephritis has no impact on maternal or fetal outcomes[14–16]. We provided the data of LN on pregnancy outcome in a large group of SLE patients in a tertiary center. Based on our results, lupus nephritis was another risk factor for adverse maternal outcomes, such as SLE flares, hypertension, and anemia, but it was not significant association with adverse fetal outcomes.

Neonatal lupus and two neonatal malformations are still challenging to physicians. There are two neonatal lupus and two fetal heart malformations (one tetralogy of Fallot, and one atrial septal defect). The neonatal with tetralogy of Fallot died at the second day after birth. Neonatal lupus and neonatal heart disease do not appear to be significantly associated with the new-onset lupus during pregnancy or LN pregnancies, which may be associated with the anti-Ro/SSA and anti-La/SSB[42,43] and needs further investigation.

There are several limitations to our study. Firstly, as a retrospective investigation, some patients were referred to our hospital in the midst of their pregnancy and those patients’ information at the starting of pregnancy were lacking. Secondly, our single-center study may have bias. Our patients with increased lupus activity during pregnancy may be due to the major tertiary hospital receiving patients from other community hospitals. So, it should be noted that our patients may not represent the general population well.

In conclusion, pregnancies can be successful in most women with pre-existing SLE. However, the new-onset of lupus during pregnancy generally have poor prognosis, with high maternal and fetal morbidity and mortality. Furthermore, a history of lupus nephritis could predict adverse maternal outcomes. To obtain favorable pregnancy outcomes, lupus quiescence, no significant proteinuria and satisfactory blood pressure control should be achieved before conception and regularly monitored by experienced obstetricians, nephrologist and rheumatologists is needed.

## Supporting Information

S1 File The data of new onset SLE group and pre-existing SLE group. (SAV)

S2 File The data of SLE group and lupus nephritis group. (SAV)
